# Supplementary material for: Production of seedable Amyloid-β peptides in model of prion diseases upon PrPSc-induced PDK1 overactivation
Source: Nat Commun. 2019 Aug 1;10:3442. doi: 10.1038/s41467-019-11333-3 (PMC6672003; doi:10.1038/s41467-019-11333-3)
Supplement: Supplementary file 1 — Supplementary Information [file 41467_2019_11333_MOESM1_ESM.pdf]

Supplementary Information

Production of seedable Amyloid-β peptides in prion diseases upon PrP^{Sc}-induced PDK1 overactivation

Ezpeleta et al.

Supplementary Fig. 1

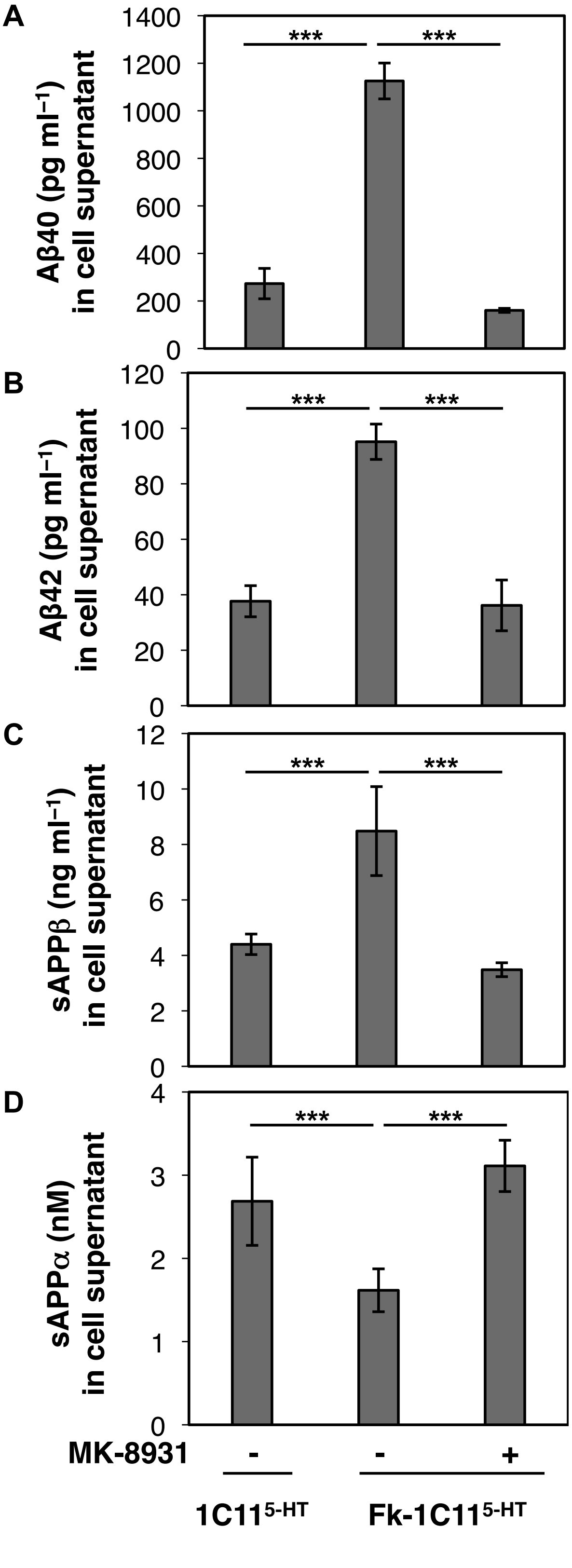

Supplementary Figure 1: Prion infection engages APP towards the β-amyloidogenic pathway. Concentrations of Aβ40 (**A**), Aβ42 (**B**), sAPPβ (**C**), and sAPPα (**D**) in the culture medium of uninfected serotonergic $1C11^{5\text{-}HT}$ and Fk-infected $1C11^{5\text{-}HT}$ neuronal cells (Fk-$1C11^{5\text{-}HT}$) treated or not with the BACE-1 inhibitor MK-8931 (1 μM) for 1h, deduced from LC-MS/MS analyses. Values are means ± sem of six independent experiments. Data were analyzed using the two-tail Student t test. ***denotes p<0.001.

Supplementary Fig. 2

A

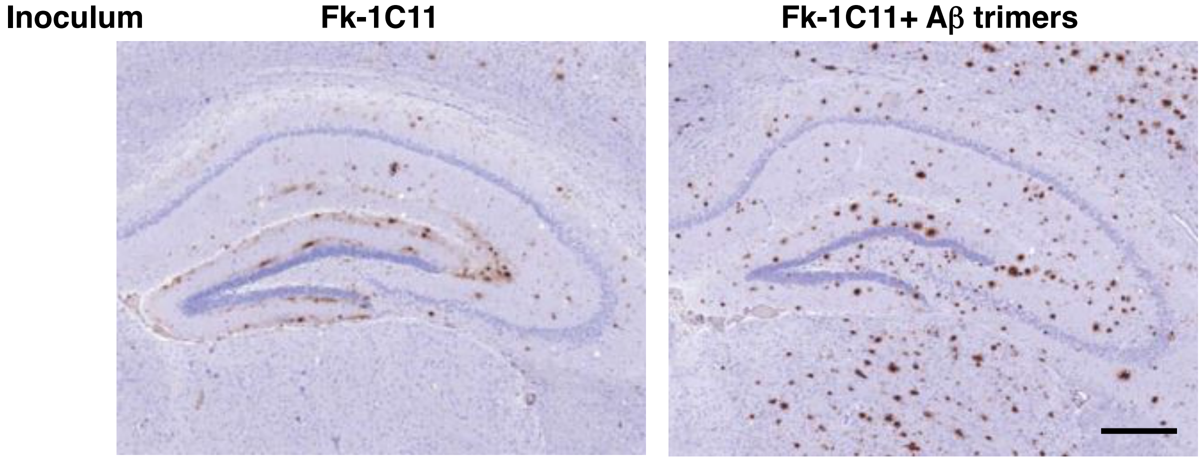

B

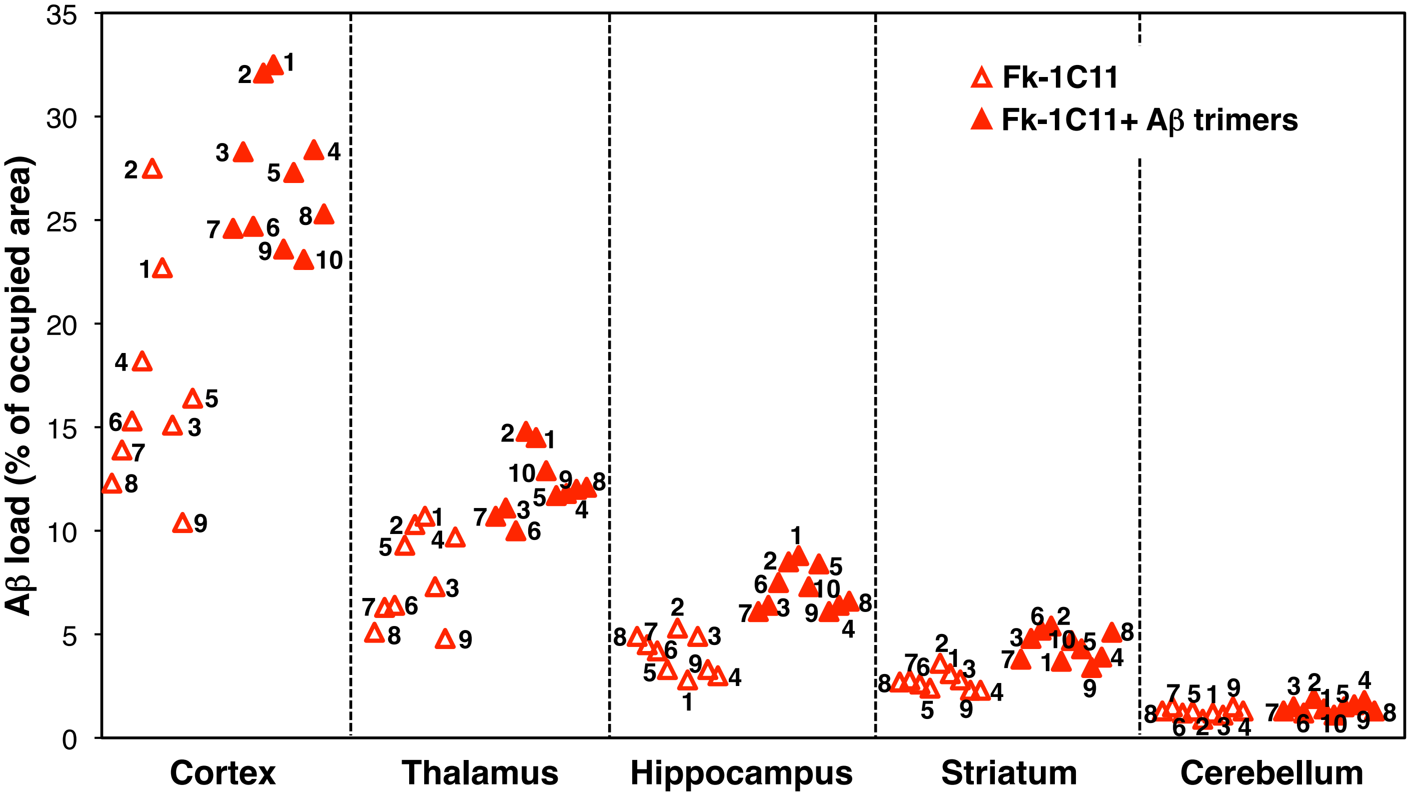

Supplementary Figure 2: Histological and quantification analyses of Aβ deposition in the brain of APP23 mice co-inoculated with PrP^{Sc} and seeds of Aβ. (**A**) Representative images of 4G8-positive Aβ plaques in the brain of APP23 mice injected with Fk-1C11-derived inocula containing low levels of Aβ seeds (Fk-1C11, mouse n° 8) or supplemented with synthetic human Aβ trimers (Fk-1C11+Aβ trimers, mouse n° 7). Scale bar = 500 µm . (**B**) β-amyloid load (% of occupied area) in the cortex, thalamus, hippocampus, striatum and cerebellum of prion-infected APP23 mice. *Open triangles*: mice injected with Fk-1C11-derived inocula. *Close triangles*: mice injected with Fk-1C11-derived inocula supplemented with Aβ trimers.

Supplementary Table 1: APP23 mice positive for β-amyloid deposition measured by PET imaging after PIB injection along prion infection.

		number of animals with ^{11}C-PIB retention/total number in each group (month post-inoculation)							
**inoculum**	**Aβ trimers**	**1 mpi**	**2 mpi**	**3 mpi**	**4 mpi**	**5 mpi**	**6 mpi**	**7 mpi**	**8 mpi**
1C11	-	0/10	0/10	0/10	0/10	0/10	0/10	0/10	0/10
	+	0/10	0/10	0/10	0/10	0/10	0/10	0/10	0/10
Fk-1C11	-	0/10	0/10	1/10	1/10	1/10	2/10	1/5<br>† 5/10	† 9/10
	+	0/10	3/10	4/10	4/10	8/10	†10/10	†10/10	†10/10
APP^{null}-Fk1C11	-	0/10	0/10	0/10	0/10	0/10	0/10	1/10	1/10
	+	0/10	0/10	0/10	1/10	2/10	2/10	† 2/10	† 2/10

†: dead

Supplementary Table 2: Impact of PDK1 inhibition on β-amyloid deposition in prion-infected APP23 mice.

			number of animals with ^{11}C-PIB retention/total number in each group (month post-inoculation)							
**inoculum**	**Aβ trimers**	**BX912**	**1 mpi**	**2 mpi**	**3 mpi**	**4 mpi**	**5 mpi**	**6 mpi**	**7 mpi**	**8 mpi**
Fk-1C11	-	-	0/10	0/10	1/10	1/10	1/10	2/10	1/5<br>† 5/10	† 9/10
	-	+	0/10	0/10	0/10	0/10	0/10	0/10	0/10	†3/10
	+	-	0/10	3/10	4/10	4/10	8/10	†10/10	†10/10	†10/10
	+	+	0/10	0/10	0/10	0/10	0/10	1/10	3/8<br>† 2/10	1/6<br>† 4/10
APP^{null}-Fk1C11	-	-	0/10	0/10	0/10	0/10	0/10	0/10	1/10	1/10
	-	+	0/10	0/10	0/10	0/10	0/10	0/10	1/10	2/10
	+	-	0/10	0/10	0/10	1/10	2/10	2/10	† 2/10	† 2/10
	+	+	0/10	0/10	0/10	0/10	0/10	1/10	2/10	2/10

†: dead
