# Supplementary material for: Production of seedable Amyloid-β peptides in model of prion diseases upon PrPSc-induced PDK1 overactivation
Source: Nat Commun. 2019 Aug 1;10:3442. doi: 10.1038/s41467-019-11333-3 (PMC6672003; doi:10.1038/s41467-019-11333-3)
Supplement: Supplementary file 3 — Reporting Summary [file 41467_2019_11333_MOESM3_ESM.pdf]

nature research

Corresponding author(s): DBPR NCOMMS-18-33195A

Last updated by author(s): Jun 20, 2019

Reporting Summary

Nature Research wishes to improve the reproducibility of the work that we publish. This form provides structure for consistency and transparency in reporting. For further information on Nature Research policies, see Authors & Referees and the Editorial Policy Checklist.

Statistics

For all statistical analyses, confirm that the following items are present in the figure legend, table legend, main text, or Methods section.

n/a	Confirmed	
☐	☒	The exact sample size (*n*) for each experimental group/condition, given as a discrete number and unit of measurement
☐	☒	A statement on whether measurements were taken from distinct samples or whether the same sample was measured repeatedly
☐	☒	The statistical test(s) used AND whether they are one- or two-sided<br>*Only common tests should be described solely by name; describe more complex techniques in the Methods section.*
☒	☐	A description of all covariates tested
☒	☐	A description of any assumptions or corrections, such as tests of normality and adjustment for multiple comparisons
☐	☒	A full description of the statistical parameters including central tendency (e.g. means) or other basic estimates (e.g. regression coefficient) AND variation (e.g. standard deviation) or associated estimates of uncertainty (e.g. confidence intervals)
☒	☐	For null hypothesis testing, the test statistic (e.g. *F*, *t*, *r*) with confidence intervals, effect sizes, degrees of freedom and *P* value noted<br>*Give P values as exact values whenever suitable.*
☒	☐	For Bayesian analysis, information on the choice of priors and Markov chain Monte Carlo settings
☒	☐	For hierarchical and complex designs, identification of the appropriate level for tests and full reporting of outcomes
☒	☐	Estimates of effect sizes (e.g. Cohen's *d*, Pearson's *r*), indicating how they were calculated

Our web collection on statistics for biologists contains articles on many of the points above.

Software and code

Policy information about availability of computer code

Data collection	No software was used.
Data analysis	The Kaleidagraph software v4.01 (Synergy Software, Reading, PA, USA) and the GraphPad Prism software v7 (San Diego, CA, USA) were used for data analysis and presentation.

For manuscripts utilizing custom algorithms or software that are central to the research but not yet described in published literature, software must be made available to editors/reviewers. We strongly encourage code deposition in a community repository (e.g. GitHub). See the Nature Research guidelines for submitting code & software for further information.

Data

Policy information about availability of data

All manuscripts must include a data availability statement. This statement should provide the following information, where applicable:

- Accession codes, unique identifiers, or web links for publicly available datasets
- A list of figures that have associated raw data
- A description of any restrictions on data availability

The 1C11 neuronal stem cell line is available upon requests.

Field-specific reporting

Please select the one below that is the best fit for your research. If you are not sure, read the appropriate sections before making your selection.

☒ Life sciences ☐ Behavioural & social sciences ☐ Ecological, evolutionary & environmental sciences

Life sciences study design

All studies must disclose on these points even when the disclosure is negative.

Sample size	No sample size calculation was performed. Sample sizes were chosen similar to those previously reported in our papers in Nature Medicine (Pietri et al., 2013, PMID: 23955714) and PloS Pathogens (Alleaume-Butaux et al., 2015, PMID: 26241960).
Data exclusions	No data were excluded throughout the study.
Replication	In our hands, all attempts at replication were successful.
Randomization	All allocations were random.
Blinding	Investigators were blinded to group allocation during data collection.

Reporting for specific materials, systems and methods

We require information from authors about some types of materials, experimental systems and methods used in many studies. Here, indicate whether each material, system or method listed is relevant to your study. If you are not sure if a list item applies to your research, read the appropriate section before selecting a response.

Materials & experimental systems

n/a	Involved in the study	
☐	☒	Antibodies
☐	☒	Eukaryotic cell lines
☒	☐	Palaeontology
☐	☒	Animals and other organisms
☒	☐	Human research participants
☒	☐	Clinical data

Methods

n/a	Involved in the study	
☒	☐	ChIP-seq
☒	☐	Flow cytometry
☒	☐	MRI-based neuroimaging

Antibodies

Antibodies used	As stated in the Methods section, Aβ42 and Aβ40 were serially immunoprecipitated from the samples using C-terminal specific antibodies (NB300-225 from Novus Biologicals and ABIN363343 from Antibodies online, Atlanta, GA, USA). Sha31 antibody (SPI-Bio, Berthin Bioreagent, Montigny Le Bretonneux, France, Cat n° A03213) was used to detect PrP. 4G8 antibody (SIG-39220, Covance,Princeton NJ USA) was used for detecting Aβ in mouse brain slices.<br>References:<br>Aβ40/Aβ42: Pradines et al. Cell Death and Diseases (2013), 4:e456. PMID: 23303130<br>Sha31: Morel et al., JBC (2004), 279: 30143-9, PMID: 15140886; Feraudet et al., JBC (2005), 280: 11247-11258, PMID: 15618225.<br>4G8: Pietri et al., Nat Med (2013), 19(9):1124-31. PMID: 23955714.
Validation	All the antibodies used in this study were validated by the manufacturers for specific detection of the antigen, mouse reactivity and western-blot, immunoprecipitation and ELISA applications.

Eukaryotic cell lines

Policy information about cell lines

Cell line source(s)	The 1C11 neuronal stem cell line was established by Pr. Odile Kellermann at the Pasteur Institute (Paris, France) in 1990 (Buc-Caron, PNAS (1990), 87:1922-6, PMID: 2155426).
Authentication	The 1C11 cell line used was not autenthicated.
Mycoplasma contamination	The 1C11 cell line and their prion-infected derivatives were tested negative for mycoplasma contamination.
Commonly misidentified lines<br>(See ICLAC register)	

The 1C11 cell line is a neuronal stem line endowed with the capacity to differentiate either into serotonergic and noradrenergic neurons (Mouillet-Richard, JBC, 2000, PMID: 10734054) that supports prion replication (Mouillet-Richard, JBC, 2008, PMID: 18617522). Since 1990, this cell line was instrumental to decipher some basic mechanisms of the neuronal differentiation (Mouillet-Richard, JBC, 2000, PMID: 10734054; Boucquey, J Neurochem, 2006, PMID: 17029606), the adaptive response of bioaminergic neurons to antidepressants (Launay, FASEB J, 2006, PMID: 16940156; Baudry, Science, 2010, PMID: 20847275; Kittler, Eur J. Pharmacol, 2010, PMID: 20006597; Launay, Trans Psy, 2011, PMID: 22833211; Matthaus, Eur Neuropsy, 2016, PMID: 27665061), the role of cellular prion protein in neurons (Mouillet-Richard, Science, 2000, PMID: 10988071; Schneider, PNAS, 2003, PMID: 14597699; Loubet, FASEB, 2012, PMID: 22038049; Ezpeleta, Scientific Reports, 2017, PMID: 28794434), mechanisms of neurodegeneration associated with prion and Alzheimer's diseases (Pietri, JBC, 2006, PMID: 16864581; Mouillet-Richard, JBC, 2008, PMID: 18617522; Pietri et al. Nat. Med. 2013, PMID: 23955714; Pradines, Cell death and disease, 2013, PMID: 23303130; Alleaume-Butaux, Plos Pathogens, 2015, PMID: 26241960), or toxic mechanisms of pollutants (Miozo, Cell Stress and Chap, 2018, PMID: 28712054).

Animals and other organisms

Policy information about studies involving animals; ARRIVE guidelines recommended for reporting animal research

Laboratory animals	As stated in the Methods section, C57Bl/6J mice (8 weeks-old) and hemizigous APP23 mice with the K670M/N671L double mutation on the human APP gene (3 months-old) were infected by prions. For APP23 mice, as there is a gender effect on the onset of Aβ pathology, only females were used in this study.
Wild animals	The study did not involve wild animals.
Field-collected samples	The study did not involve samples collected from the field.
Ethics oversight	All animal procedures were approved by the Animal Care and Use Committee at Basel University (Switzerland) and by the Comité Régional d'Ethique en Matière d'Expérimentation Animale de Strasbourg (France) with number CEEA35 ref AL/01/01/01/13.

Note that full information on the approval of the study protocol must also be provided in the manuscript.
